# Comparison of the Effectiveness of Whole Body Vibration in Stroke Patients: A Meta-Analysis

**DOI:** 10.1155/2018/5083634

**Published:** 2018-01-02

**Authors:** Yoo Jung Park, Sun Wook Park, Han Suk Lee

**Affiliations:** ^1^Department of Physical Therapy, Samsung Seoul Hospital, 81 Irwon-ro, Gangnam-Gu, Seoul 06351, Republic of Korea; ^2^Department of Physical Therapy, Faculty of Health Science, Eulji University, 212 Yangji-dong, Sujeong-gu, Seongnam, Gyeonggi-do 461-713, Republic of Korea

## Abstract

**Objectives:**

The goals of this study were to assess the effectiveness of WBV (whole body vibration) training through an analysis of effect sizes, identify advantages of WBV training, and suggest other effective treatment methods.

**Methods:**

Four databases, namely, EMBASE, PubMed, EBSCO, and Web of Science, were used to collect articles on vibration. Keywords such as “vibration” and “stroke” were used in the search for published articles. Consequently, eleven studies were selected in the second screening using meta-analyses.

**Results:**

The total effect size of patients with dementia in the studies was 0.25, which was small. The effect size of spasticity was the greatest at 1.24 (high), followed by metabolism at 0.99 (high), balance, muscle strength, gait, and circulation in the decreasing order of effect size.

**Conclusions:**

The effect sizes for muscle strength and balance and gait function, all of which play an important role in performance of daily activities, were small. In contrast, effect sizes for bone metabolism and spasticity were moderate. This suggests that WBV training may provide a safe, alternative treatment method for improving the symptoms of stroke in patients.

## 1. Introduction

Stroke rehabilitation is a process through which patients with disabilities as a result of stroke manage to resume activities of daily living and reestablish their normal lifestyle through a learning process. It also aims to assist patients in gaining better understanding of their condition, adapting to difficulties they experience due to their disabilities, and preventing secondary complications [1]. Typical disabilities that follow stroke include muscle weakness, abnormal muscle stress, and dystonia. These disabilities not only limit daily activities but also affect the balancing ability and gait function [2].

To a large extent, research has been conducted with the aim of resolving these stroke-related problems. Recently, whole body vibration (WBV) has been heavily researched as a way to improve muscle function, muscle strength, and gait function in stroke patients [3, 4]. WBV training involves standing or making vigorous movements on a vibration platform placed on a static surface. In previous studies, WBV training was suggested as a potential method to improve physical functions. It was also suggested that WBV improves muscle function and balance by increasing muscle strength.

Therapies that involve WBV exercises have been on the rise recently; however, only a few studies have compared WBV therapy with other treatment modalities. Therefore, this study aims to compare the effects of WBV treatment using meta-analysis. Further, the purpose of this study is to assess the effectiveness of WBV training through an analysis of effect sizes, identify advantages of WBV training, and suggest other effective treatment methods.

## 2. Material and Method

### 2.1. Research Question

The purpose of this systemic review was determined according to PICO (patient, intervention, comparison, and outcome). In this review, the patient (P) was defined as a person having “stroke.” The intervention (I) was defined in the experimental group that underwent WBV training (static activities and vigorous exercise). The experimental group was compared (C) to the control group that did not undergo WBV training. The outcome (O) was defined as changes in motor functions and body structure. This study investigated effect sizes of WBV training on different variables and determined the ones on which WBV had the greatest effect.

### 2.2. Selection and Collection of Articles to Be Analyzed

**Figure 1:**
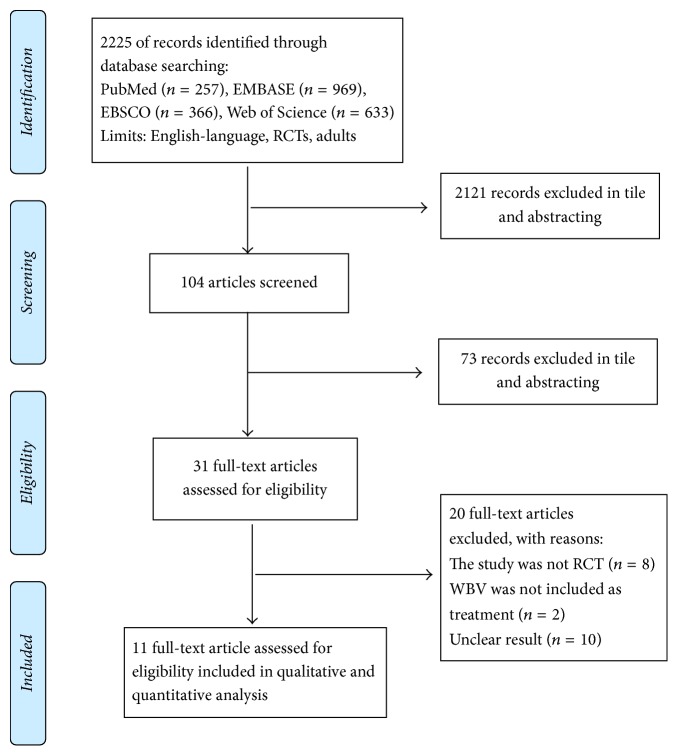
Flow diagram of studies included.

Articles related to WBV were searched for in 5 databases including EMBASE, PubMed, EBSCO, and Web of Science for inclusion in the meta-analysis. The criteria for selecting a dissertation were as follows: studies involving clinical diagnosis of stroke and its treatment in a randomized controlled clinical trial. The language was limited to English. “Vibration” and “stroke” were used as keywords in order to minimize the number of articles that would be missed when searching solely with the keyword, “WBV.” Using these keywords, a total of 2225 studies were initially selected, after which we excluded the ones that did not report sufficient statistics. The exclusion criteria were reviewed by examining the title and abstract of the papers with respect to the subject, while the main text and the theory of research were excluded from the analysis. We also excluded research that was difficult to classify after reviewing the design methods of each analyzed study. Of the 2225 initially selected articles, 2121 were excluded. Upon reviewing the titles and abstracts of the selected studies, 73 were further excluded and 31 were selected based on the research topic. Within these, 20 studies were excluded owing to the following reasons: eight studies were not randomized controlled trials, two articles did not investigate general vibration training, and 10 papers showed ambiguous results that did not provide sufficient statistical data in relation to the meta-analysis. Finally, 11 studies were selected and the characteristics of PRISMA flow chart were summarized (Figure 1, Table 1). The selected studies analyzed the treatment effects of WBV in stroke patients. The number of patients used in the final analysis was 4,413. A detailed description of the included individual studies is presented in Table 1. A review of the methodologies used in the selected articles revealed that most of them used a one-group pretest-posttest design (Table 2).

### 2.3. Data Processing

With the agreement of all members of the research team, the author names, published year, publication type, research model, study participants, assessment tools, program type, and program effectiveness were recorded for data coding. A physical therapist and a meta-analysis specialist performed the coding. Conflicts in opinions were resolved through negotiation and opinions of a physiotherapy professor. The credibility and consistency of people involved in coding were not calculated.

### 2.4. Data Extraction

A CMA software specialized in meta-analysis was used for data analysis. In order to interpret the effect sizes obtained from the meta-analysis, Cohen and Wolf's standard was used. According to Cohen [16], an effect is small if it is less than 0.2, moderate if it is 0.5, and large if it is greater than 0.8.

### 2.5. Quality Assessment

Using the PEDro database's method of analysis, a quality assessment of randomized controlled trial articles was performed. The PEDro scale determines the scientific validity of clinical trials (9-10 = excellent, 6–8 = good, 4–6 = fair, and <4 = poor). Studies of excellent or good qualities with a sample size greater than or equal to 50 were considered as level 1 evidence [17] (Table 3).

## 3. Results

### 3.1. Homogeneity Test and Total Effect Size

**Figure 2:**
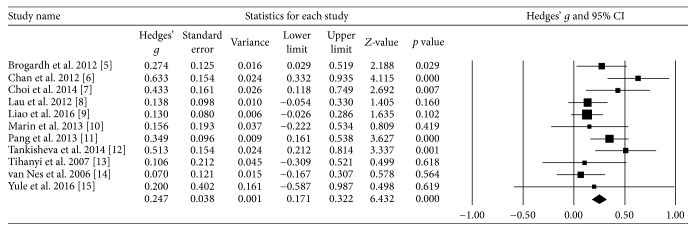
Homogeneity test.

Assuming that results of each study were based on one homogeneous population, a homogeneity test with a fixed-effects model was performed. The *Q* value was 18.02, verifying that the studies were performed on homogeneous population. Considering each subject's result as one unit and using a random effects model, a “standardized mean difference” effect size (*d*) was calculated. The obtained total effect size of WBV was 0.25, and the 95% confidence interval was 0.17~0.32 (Table 4, Figure 2). Since the effect size of WBV on stroke was close to 0.2, it was interpreted that WBV has a “small effect size.”

### 3.2. Publication Bias Assessment

Publication biases were assessed to validate the results of meta-analysis using three different methods. A type of sensitivity analysis was performed using Duval and Tweedie's [18] trim-and-fill method. Since the correction values of articles and the observed values were identical, it was difficult to conclude if publication bias was present (Table 5).

### 3.3. Effect Size according to Treatment Effectiveness

As presented in Table 5, the effect size of spasticity was the largest at 1.24, followed by bone metabolism at 0.99, balance, muscle strength, gait, and cardiac function, in decreasing order of effect size (Table 6).

### 3.4. Effect Size at Different Vibration Frequencies

**Figure 3:**
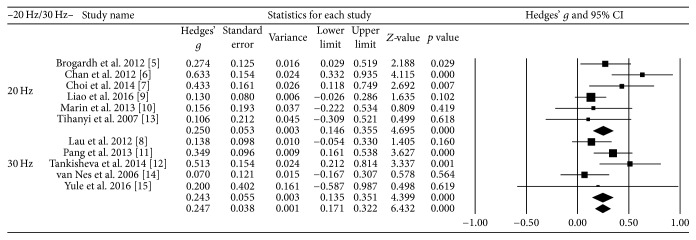
Effect size at different vibration frequencies.

Vibration frequencies below 20 Hz were considered low frequencies and those over 30 Hz were considered high frequencies. The effect size was 0.25 at high frequency and 0.24 at low frequency; therefore, there was no significant difference in the effect sizes between high and low frequency (Figure 3).

### 3.5. Effect Size according to the Time Lapse after the Onset of Stroke

**Figure 4:**
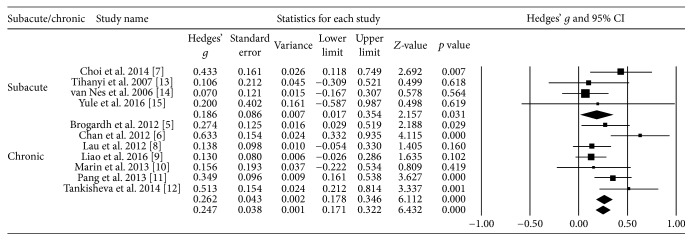
Effect size according to the time lapse after the onset of stroke.

The effect size was 0.26 when time lapse after the onset of stroke was over a year (chronic) and 0.19 when the time lapse was under one year (acute/subacute). The effect size of acute/subacute stroke was close to 0.2, which signifies a small effect size. In contrast, the effect size was relatively large for chronic stroke (Figure 4).

### 3.6. Effect Size according to the Treatment Period

**Figure 5:**
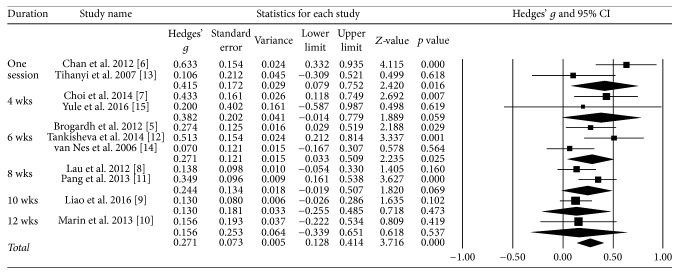
Effect size according to the treatment period.

The effect size was 0.42 for one session and 0.4 for four weeks of therapy; both effect sizes were moderate (Figure 5).

### 3.7. Effect Size according to the Number of Treatments per Week

**Figure 6:**
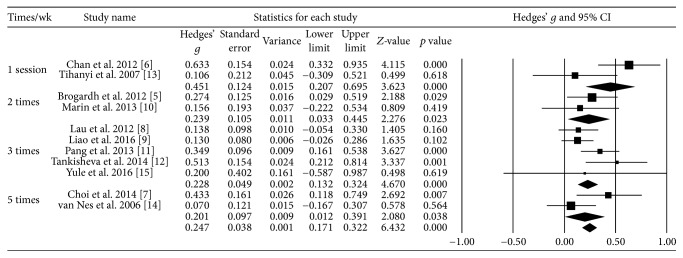
Effect size according to the number of treatments per week.

The effect size was 0.45 for one session and the effect size for the other times was 0.2 (Figure 6).

### 3.8. Changes per Published Year

**Figure 7:**
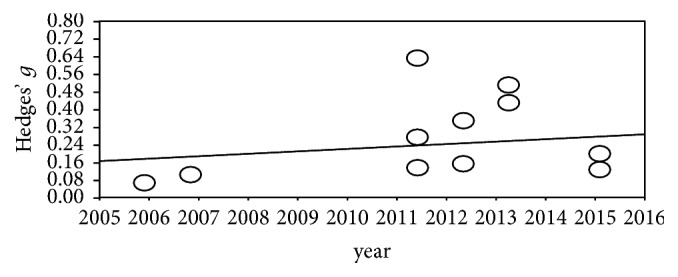
Changes per published year.

Research on WBV for stroke patients started recently, and the number of studies is gradually increasing every year (Figure 7).

## 4. Discussion

This study was conducted to investigate the effectiveness of WBV through a meta-analysis of numerous studies on WBV therapy that were published recently. According to Lee et al. [2], muscle dystrophy, muscle tone, and loss of sensation in the aftermath of stroke affect the ability to function and walk. In this study, we analyzed the magnitude of these effects on muscle strength, locomotion, muscular dystrophy, and balance and analyzed the effects of bone density and circulation in order to prevent secondary complications. Previous meta-analyses have investigated the effects of WBV on balance, gait function, and limb movement [19], activity and participation after stroke [20], muscle strength, proprioceptive sense, and quality of life [21], and muscle stress [22]. However, in a meta-analysis by Lu et al. [23], WBV did not have significant effects on muscle strength, balance, and gait function. Further, this suggests a need for investigations based on the therapeutic efficacy of WBV in stroke patients.

In our meta-analysis, the total effect size was 0.25, which signifies a small effect size. However, when we evaluated the therapeutic efficacy of WBV for stroke symptoms, the effect size for spasticity was the largest at 1.24, followed by bone turn over test at 0.99, balance, muscle strength, gait function, and circulation in the decreasing order of effect size. Evaluation of spasticity comprised MAS, ATR, and so forth; muscle strength was isometric and isometric exercise was also evaluated. The evaluation comprised TUG, 10 MWT, and so forth, and the balance was between BBS, MFRT, and so forth. The goal was to measure bone metabolism using CTx and BAP, while cardiac function was evaluated by measuring HR and BP. According to the results of this study, WBV was more effective for spasticity that affects gait function than for gait function itself and muscle strength. This is consistent with the results of a study by Chan et al. [6], who reported that WBV reduced ankle plantarflexion spasticity in chronic stroke patients and therefore would be useful in gait function improvement. Moreover, they reported that WBV did improve gait function and would improve movements and movement speed. Another previous study also reported that a reduction in ankle plantarflexion spasticity affects gait function, limb movement, and movement speed [24, 25].

Even if bone metabolism does not affect gait function and risk of falls, it may prevent secondary physical problems that occur upon falling among stroke patients. Pang et al. [11] used a bone turnover test to evaluate the effects on bone mineral density. While no significant differences in the effects between the experimental and control groups were noted, it was suggested that the treatment period be extended or the therapeutic intensity be increased. Garnero et al. [26] also reported that the level of bone turnover could be useful for osteoporosis risk assessment. Considering that the majority of stroke patients are at an advanced age and have a high risk of falls due to the reduced control of their bodies, it is expected that WBV may be an effective treatment for bone weakening.

While Lau et al. [8] reported that WBV had no effect on risk of falls or motor functions, their study focused on self-efficacy of falls, which is related to balance, postural control, mobility, and muscle strength and balance. Although it cannot be definitively concluded that WBV directly improves motor functions and thereby prevents falls, WBV may certainly prevent secondary problems that occur in patients who had strokes due to an accidental fall. In this study, the effect size was small for balance, gait function, and muscle strength.

With regard to balance and muscle strength, Tihanyi et al. [13] reported that WBV was effective in increasing voluntary muscle strength, which further helped balancing and gait function. Lau et al. [8] reported that WBV eliminated risk of falls and enhanced motor functions in stroke patients during leg exercises. Regarding balance, van Nes et al. [14] reported improvements in performance of activities of daily life and balance in the WBV group compared to controls after a 12-week program. As reported by Choi et al. [7], WBV improved sitting balance and was suggested as an effective training method to improve balance. It was also reported that WBV was helpful for stimulation of the vestibular system, posture improvement, and posture correction [27] as well as postural sway enhancement [28].

However, WBV in our analysis had a small effect compared to these individual studies. The results of our study are consistent with those of Brogardh et al. [5], in which WBV had a small effect on balance and gait function improvement. They are also consistent with the results obtained by Marin et al. [10], in which there was no significant difference in muscle strength and balance between the WBV and control groups.

Similarly, Yule et al. [15] concluded that WBV does not effectively improve physical functions related to muscle strength and balance that are related to walking and activities of daily life; however, they suggested that WBV is a safe method to improve spasticity, which is related to safety and sitting balance. Likewise, Liao et al. [9] and Tankisheva et al. [12] reported that WBV is a safe therapy that can be used to improve physical functions, structure, activity, and muscle gain. Since WBV safely reduces spasticity and has an effect on bone mineral density, it may be expected to prevent secondary problems caused by accidental falls. Although these analyses compared the effects of vibration frequencies, there was no difference in the magnitude of effects according to frequency. Further, there were no significant differences in the amount of effects caused by the period of stroke; however, it appeared somewhat higher in the chronic period. This implies that WBV is therapeutic for stroke when considered in terms of spontaneous recovery. There was no difference in the effectiveness on the basis of number of sessions and weeks of treatment. Because our meta-analysis studies lacked a sufficiently large number of studies, we need to further evaluate studies on WBV for stroke. A small sample size was used in this study to establish indisputable evidence. In future, a higher number of studies on effectiveness according to the timing of stroke are required.

## 5. Conclusion

This study investigated effect sizes of WBV training therapy for stroke patients through a meta-analysis. The number of analyzed articles was perhaps too small because studies that included subjects without a diagnosis of stroke were excluded, several studies investigated the effects of intervention qualitatively, and several others were nonrandomized controlled studies or did not have control groups.

The purpose of our study was to verify the efficacy of WBV training as a novel approach to stroke treatment and suggest more effective treatment methods. Effect sizes from WBV studies with a pretest-posttest design and a control group were obtained, and the total effect size was small. The effect sizes for muscle strength and balance and gait function, all of which play an important role in performance of daily activities were small. In contrast, effect sizes for bone metabolism and spasticity were moderate. WBV training is a safe therapeutic method for improving symptoms in stroke patients.

## Figures and Tables

**Table 1:** Characteristic of included trials.

Study	Number of participants analyzed (*E*/*C*)	Mean age (*E*/*C*)	Start REH intervention (*E*/*C*)	Duration of prog.	Time
Brogardh et al. 2012 [5]	16/15	61.3 ± 8.5/63.9 ± 5.8	37.4/33.1 m	2/week*∗*6	45 min
Chan et al. 2012 [6]	15/15	56.07 ± 11.04/54.93 ± 7.45	30.4/38.87 m	One session	
Choi et al. 2014 [7]	15/15	62.8 ± 9/65.1 ± 15.7	13/12.6	5/w*∗*4	15 min
Lau et al. 2012 [8]	41/41	57.3 ± 11.3/57.4 ± 11.1	4.6/5.3 y	3/w*∗*8	
Liao et al. 2016 [9]	28/28	59.8 ± 9.1/60.8 ± 8.3	8.5/9.0 y	3/w*∗*10	12 min
Marin et al. 2013 [10]	11/9	62.3 ± 10.6/64.4 ± 7.6	4.3/4.3 y	12 w (17 sessions)	
Pang et al. 2013 [11]	38/38	57.3 ± 11.3/57.4 ± 11.1	4.6/5.3 y	3/w*∗*8	
Tankisheva et al. 2014 [12]	6/7	57.4 ± 13/65.3 ± 3.7	7.71/5.28 y	3/w*∗*6	30 min
Tihanyi et al. 2007 [13]	8/8	58.2 ± 9.4	27.2 ± 10.4 d	One session	
van Nes et al. 2006 [14]	27/26	59.7 ± 12.3/62.6 ± 7.6	38.9/34.2 d	5/w*∗*6	
Yule et al. 2016 [15]	4/2	50.5 ± 14.5/39 ± 2	6 m–5 y	3/w*∗*4	15 min

**Table 2:** Review of the studies.

Study	Outcome measures	Type of intervention	control	frequency	amplitude
Brogardh et al. 2012 [5]	Muscle tone: MAS Balance: BBS Muscle strength measurements Gait performance: TUG, 10 MGS, 6 MWT Participation: SIS	Standing barefoot on the platforms in a static position with the knee flexed 45° 60°	vibrating platform with an amplitude of 0.2 mm	25	3.75

Chan et al. 2012 [6]	Ankle spasticity: MAS, deep tendon reflex, VAS Gait performance: TUG, 10 MWT, cadence Foot pressure	Positioned on the platform in a semisquatting position with buttock support and were kept in an upright position with even weight distribution on both feet	same procedure, No WBV	12	4

Choi et al. 2014 [7]	Static sitting balance: COP Dynamic sitting balance: MFRT	Task oriented training + WBV (1) Sitting alone at a table and correcting body alignment (2) Reaching in different directions for objects located beyond arm's length using the nonparetic side (3) Reaching in different directions for objects located beyond arm's length using the paretic side (4) A bilateral reaching task	task oriented training	15–22	0–5.8

Lau et al. 2012 [8]	Balance: BBS Dynamic postural control: LOS Muscle strength measurements (70°) Gait performance: 10 MWT, 6 MWT Fall-related self-efficacy ABC	Side-to-side weight shift, semisquat, forward and backward weight shift, forward lunge, standing on one leg, deep squat	same platform, No WBV	20–30	0.44–0.6

Liao et al. 2016 [9]	Muscle tone: MAS Balance: Mini BESTest Gait performance: TUG, 6 MWT Fall-related self-efficacy ABC	Dynamic weight shift side to side, dynamic deep squat, dynamic forward and backward weight shift, static semisquat	same platform, No WBV	20	1

Marin et al. 2013 [10]	Balance: BBS Muscle strength: thickness of RF, VL, MG in both legs Maximum isometric knee extension strength	Standing on a vibration platform with knee flexion of 30°	same position, No WBV	5–21	4–6

Pang et al. 2013 [11]	Bone turnover markers Spasticity: MAS, VAS Muscle strength: knee peak power	Side-to-side weight shift, semisquat, forward and backward, weight shift, forward lunge, standing on one leg, deep squat	same platform, No WBV	20–30	0.44–0.6

Tankisheva et al. 2014 [12]	Muscle tone: MAS Muscle strength measurements: isokinetic knee extension in both legs (60°/s) Isokinetic knee flexion in both legs (60°/s) Isometric knee extension in both legs Isometric knee flexion in both legs Isokinetic knee extension in nonparetic leg (240°/s) Isokinetic knee flexion in nonparetic leg (240°/s) SOT Equilibrium scores	Standing on their toes, knee flexion of 50–60, knee flexion of 90°, wide-stance squat, one-legged squat	No	35, 40	1.7, 2, 5

Tihanyi et al. 2007 [13]	EMG: Maximum isometric knee extension torque Maximum eccentric knee extension torque Rate of torque development Maximal voluntary eccentric torque at 60° of knee flexion Coactivation quotient of BF during Isometric knee extension Coactivation quotient of BF during eccentric knee extension	Standing the platform with knees slightly flexed at 40° and shifting body mass to the paretic leg	same platform, No WBV	20	5

van Nes et al. 2006 [14]	Balance: BBS BI Rivermead Mobility Index Trunk Control Test FAC Motricity Index Somatosensory threshold of affected leg	Standing on the platform with knees slightly flexed	Exercise therapy on music	30	

Yule et al. 2016 [15]	Pulse wave velocity Carotid to radial PTT Arterial stiffness Heart rate Blood pressure Augmentation index	Static squat stance with 70° knee flexion	No	22–26	

MAS: Modified Ashworth Scale, BBS: Berg Balance Scale, TUG: Timed Up & Go, SIS: Stroke Impact Scale, 10 MGS: 10 miters' gait speed, 6 MWT: six-minute walk test, COP: Center of Pressure; MFRT: Modified Functional Reach Test, LOS: Limit of Stability, 10 MWT: 10 miters' walk test, ABC: activities-specific balance confidence scale.

**Table 3:** Quality assessment.

	Brogardh et al. (2012)	Chan et al. (2012)	Choi et al. (2014)	Lau et al. (2012)	Liao et al. (2016)	Marin et al. (2013)	Pang et al. (2013)	Tankisheva et al. (2014)	Tihanyi et al. (2007)	van Nes et al. (2006)	Yule et al. (2016)
Eligibility criteria	Yes	Yes	Yes	Yes	Yes	Yes	Yes	Yes	Yes	Yes	Not Yet
Random allocation	Yes	Yes	Yes	Yes	Yes	Yes	Yes	Yes	Yes	Yes	Not Yet
Concealed allocation	Yes	Yes	No	Yes	Yes	Yes	Yes	Yes	Yes	Yes	Not Yet
Baseline comparability	No	Yes	Yes	Yes	Yes	Yes	Yes	Yes	Yes	Yes	Not Yet
Blinded subjects	Yes	Yes	No	No	No	No	No	No	No	No	Not Yet
Blinded therapists	Yes	No	No	No	No	No	No	No	No	No	Not Yet
Blinded assessors	Yes	Yes	No	Yes	Yes	Yes	Yes	Yes	No	Yes	Not Yet
Adequate follow-up	Yes	Yes	Yes	Yes	Yes	Yes	Yes	Yes	Yes	Yes	Not Yet
Intention-to-treat analysis	Yes	No	Yes	Yes	Yes	Yes	Yes	No	No	Yes	Not Yet
Between-group comparisons	Yes	Yes	Yes	Yes	Yes	Yes	Yes	Yes	Yes	Yes	Not Yet
Point estimators and variability	Yes	Yes	Yes	Yes	Yes	Yes	Yes	Yes	Yes	Yes	Not Yet
Total PEDro score	9	8	6	8	8	8	8	7	6	8	Not Yet
Sample size ≥ 50	No	No	No	Yes	No	No	Yes	No	Yes	Yes	No
Level of evidence	2	2	2	1	2	2	1	2	1	1	2

**Table 4:** Homogeneity test and the total effect size.

*N*	*Q*-value	*p*	*I* ^2^	Point estimate	95% CI	Standard error
11	18.02	0.00	44.5	0.25	0.17–0.32	0.04

**Table 5:** Trim-and-fill publication bias assessment.

	Studies trimmed	Point estimate	95% CI		*Q*-value
			Lower limit	Upper limit	
Observed values	-	0.25	0.17	0.32	18.02
Adjusted values	0	0.25	0.1	0.32	18.02

**Table 6:** Effect size according to treatment effectiveness.

Group	Number of studies	Point estimate	Standard error	95% CI
Balance	19	0.28	0.08	0.12–0.43
Muscle strength	40	0.16	0.05	0.07–0.25
Gait function	15	0.09	0.07	−0.06–0.24
Spasticity	3	1.24	0.23	0.76–1.7
Bone metabolism	2	0.99	0.18	0.65–1.35
Cardiac function	3	0.2	0.4	−0.59–0.99

Total	82	0.22	0.04	0.16–0.29
